# Costs of crowding for the transmission of malaria parasites

**DOI:** 10.1111/eva.12048

**Published:** 2013-02-11

**Authors:** Laura C Pollitt, Thomas S Churcher, Emma J Dawes, Shahid M Khan, Mohammed Sajid, María-Gloria Basáñez, Nick Colegrave, Sarah E Reece

**Affiliations:** 1 Institute of Evolutionary Biology, University of EdinburghEdinburgh, UK; 2 Center for Infectious Disease Dynamics, Pennsylvania State UniversityUniversity Park, PA, USA; 3 Department of Infectious Disease Epidemiology, Imperial College LondonLondon, UK; 4 Leiden Malaria Research group, Leiden University Medical CenterThe Netherlands; 5 Centre for Immunity, Infection and Evolution, University of EdinburghEdinburgh, UK

**Keywords:** *Anopheles stephensi*, density dependence, disease transmission, fitness costs, life-history strategies, *Plasmodium berghei*, programmed cell death, vector-borne disease

## Abstract

The utility of using evolutionary and ecological frameworks to understand the dynamics of infectious diseases is gaining increasing recognition. However, integrating evolutionary ecology and infectious disease epidemiology is challenging because within-host dynamics can have counterintuitive consequences for between-host transmission, especially for vector-borne parasites. A major obstacle to linking within- and between-host processes is that the drivers of the relationships between the density, virulence, and fitness of parasites are poorly understood. By experimentally manipulating the intensity of rodent malaria (*Plasmodium berghei*) infections in *Anopheles stephensi* mosquitoes under different environmental conditions, we show that parasites experience substantial density-dependent fitness costs because crowding reduces both parasite proliferation and vector survival. We then use our data to predict how interactions between parasite density and vector environmental conditions shape within-vector processes and onward disease transmission. Our model predicts that density-dependent processes can have substantial and unexpected effects on the transmission potential of vector-borne disease, which should be considered in the development and evaluation of transmission-blocking interventions.

## Introduction

The density of individuals within a shared environment is a key factor in determining fitness, consequently shaping both ecological and evolutionary processes (MacArthur and Wilson 1967; Mueller et al. 1991; Bassar et al. 2010). In general, individuals at high densities are expected to experience increased mortality and lower reproductive success, due to competition for resources, apparent competition (e.g. increased density of shared predators) or direct interference competition (Begon et al. 2005). However, interactions between different forms of competition and environmental variation complicate efforts to understand the link between density dependence and evolutionary and ecological processes (Begon et al. 2005; Aboagye-Antwi et al. 2010). This is because identifying the life-history stages and demographic factors that are most sensitive to density dependence is often very difficult (Bassar et al. 2010). For parasites, this is particularly challenging because their fitness and epidemiology depend on interactions with a number of other organisms during their life cycle (co-infecting parasites, hosts, and vectors) that may also be shaped by density-dependent processes.

Density dependence has important consequences for the severity and transmission of infectious diseases (Dietz 1988; Bush and Lotz 2000; Basáñez et al. 2009). For example, the density of parasites within infections can influence the fitness of both hosts and parasites in a variety of ways, including by (i) determining the outcome of apparent competition or competition for resources; (ii) altering the survival and reproductive output of infected hosts; and (iii) affecting the efficacy of drug treatment (Mackinnon et al. 2008; de Roode et al. 2008; Luong et al. 2010; Schneider et al. 2012). However, for microparasitic vector-borne diseases, how the density of parasites within the host influences within-vector processes and onwards transmission to new hosts has generally been a neglected area of research (but see Mideo et al. 2008; Day et al. 2011; Mideo et al. 2011). Linking within-host processes with patterns of transmission requires identifying the factors that influence parasite dynamics within the vector and quantifying their implications for epidemiological parameters.

Malaria parasites provide a unique system to study the causes and consequences of density-dependent processes in the vector. This is due to well-developed laboratory model systems (e.g. Ferguson and Read 2002a; Hurd et al. 2005; Sinden et al. 2007; Cirimotich et al. 2010; Paaijmans et al. 2012) and established frameworks for modelling epidemiological patterns (e.g. McKenzie and Bossert 1997; Smith and McKenzie 2004; Hancock et al. 2009; Churcher et al. 2010; Griffin et al. 2010). However, despite over a century of research since mosquitoes were identified as malaria vectors (Ross 1897), the role of density dependence in malaria transmission is controversial. For example, there is marked variation in the intensity and prevalence of malaria infection in wild caught mosquitoes (Medley et al. 1993; Billingsley et al. 1994; Tripet et al. 2008), but the explanations for this variation, and the consequences for transmission, remain unclear. Understanding how parasite density shapes transmission is important for understanding malaria epidemiology and for the development and evaluation of transmission-blocking interventions, where the goal is generally to reduce parasite density within the vector (e.g. Miura et al. 2007; Chowdhury et al. 2009). Here, we use the model parasite-vector system *Plasmodium berghei-Anopheles stephensi,* to experimentally generate variation in infection intensity and quantify how interactions between density dependence and environmental conditions can affect the potential for parasites to transmit to new hosts. We then use our data to parameterize mathematical models predicting the consequences of these interactions for epidemiological patterns.

## Materials and methods

### Study system

The transmission of malaria parasites from an infected vertebrate host to a vector occurs when sexually differentiated stages (gametocytes) are taken up in a mosquito blood meal. Gametocytes rapidly differentiate into male and female gametes and mate within the blood meal and then undergo several developmental transitions before becoming infectious to new hosts. Density-dependent processes are hypothesized to occur at multiple points during the progression of malaria parasites through the vector (termed sporogony). First, the densities of male and female gametocytes influence mating success (Reece et al. 2008). Second, 18–20 h after gametocytes mate the resulting zygotes develop into motile ookinetes, which traverse the midgut wall and invade the epithelium of the vector. Invading ookinetes could increase vector mortality in a density-dependent way, due to damage caused directly to the midgut and by the increased potential for secondary bacterial infections to establish in the haemocoel (Dimopoulos et al. 2002; Rodrigues et al. 2010). Third, after invading the epithelium, ookinetes differentiate into oocysts. Within each oocyst, there is replication and differentiation, resulting in the production of thousands of sporozoites. Sporozoite production is an energy-costly process that may suffer from resource limitation at high oocyst densities (Carwardine and Hurd 1997). Fourth, oocysts rupture to release sporozoites which then migrate to the salivary glands. Colonization of the salivary glands may impact on vector survival and parasite transmission (Koella et al. 1998) because sporozoites may clog up salivary glands and so, increase mosquito probing behaviour and/or because parasites manipulate vectors to increase their persistence and recruitment to blood feeding (Wekesa et al. 1992; Anderson et al. 1999). Fifth, for all developmental stages in the vector, parasites are vulnerable to immune responses and the strength of these responses may depend on both the density of malaria parasites and the density of bacteria introduced when ookinetes migrate through the midgut (Meister et al. 2009; Cirimotich et al. 2010; Rodrigues et al. 2010; Mendes et al. 2011; White et al. 2011). The impact of all these processes on parasite fitness may be additive or multiplicative and may also be influenced by the environmental conditions experienced by the vector (Lambrechts et al. 2006; Fellous and Koella 2010). Although malaria transmission is determined by both the longevity of vectors and the productivity of parasites (presence and density of salivary gland sporozoites), the latter is rarely studied (but see Sinden et al. 2007), and the potential interactions between density-dependent factors are largely ignored (but see Dawes et al. 2009a; Churcher et al. 2010).

### Parasites, hosts and vectors

Here, we manipulated the density of *P. berghei* parasites infecting the mosquito *Anopheles stephensi* to systematically quantify the impact of density-dependent processes on the transmission potential of malaria parasites. In this system*,* sporozoites can be released from oocysts as early as day 14 but will have reached the salivary glands and plateaued in density by 21 days after an infected blood meal (Dawes et al. 2009b). We undertook a large-scale experiment, involving two environmental conditions (for the vector), in which we measured parasite productivity and vector survival across all stages of sporogony and for the month following sporozoites reaching the salivary glands.

We used two genotypes of *Plasmodium berghei*, Pb820cl1m1 cl1 (RMgm-164; Ponzi et al. 2009) and PbMC1-KO (RMgm-153; S. Khan & M. Sajid) both originating from the ‘high gametocyte producing’ ANKA strain. PbMC1 has been found to result in higher density infections in mosquitoes than Pb820cl1m1 cl1 (S. Khan and M. Shahid, unpublished data), which we confirmed in a pilot study (mean oocyst density per mosquito; PbMC1-KO = 376 (± 48.9); Pb820cl1m1 cl1 = 268 (± 34.6); *n* = 6 cages of 75 mosquitoes per line). The PbMC1-KO genotype is genetically modified for the deletion of metacaspase 1 (MC1). This genotype was initially generated to investigate if metacaspase 1 is essential for apoptosis of ookinetes. Previous studies have revealed no significant cell death phenotype associated with MC1 in *Plasmodium berghei* (Le Chat et al. 2007). We also find that MC1 is not essential for parasite apoptosis, as both PbMC1-KO and Pb820cl1m1 cl1 ookinetes display DNA fragmentation, a marker for apoptosis (TUNEL *in situ* cell death detection kit, Fluorescein; Roche, *n* = 5 infections per line, see Pollitt et al. 2010). For simplicity, Pb820cl1m1 cl1 is hereafter referred to as the ‘regular density’ (RD) line, and PbMC1-KO as the ‘high density’ (HD) line.

All vertebrate hosts were 8–12-week-old male MF1 mice (in-house supplier, University of Edinburgh). Mice were infected with approximately 5 x 10^7^ parasitized red blood cells of the RD or HD lines (*n* = 12 infections per line for a total of 24 mice) 3 days prior to transmission to mosquitoes. Prior to transmission, thin blood smears were made and red blood cell densities were estimated using flow cytometry (Beckman Coulter Counter). The gametocytaemia (proportion of red blood cells infected with gametocytes) and sex ratio (proportion of male gametocytes) were estimated by microscopy, and the density of gametocytes in blood meals was calculated by multiplying the red blood cell density and gametocytaemia. Each of the 24 infected mice, plus an additional 8 uninfected control mice (age- and sex-matched) were anaesthetized (1.7 parts Dormitor, 1.3 parts Vetelar in 7 parts PBS given at 4 μL/g) and each mouse was exposed to a single experimental cage of mosquitoes (see below). Any unfed mosquitoes were removed from the cages (< 5 per cage). All procedures were carried out in accordance with the UK Animals (Scientific Procedures) Act 1986.

*Anopheles stephensi* mosquito colonies were maintained under standard insectary conditions of 27 ± 1°C, 70% humidity and 12:12 light:dark cycle. Larvae were reared in plastic trays at a density of 250/1.5L of distilled water and on days 11–13 after egg hatching, pupae were collected and transferred to large emergence cages (1 cage per day) with *ad libitum* access to 10% glucose solution supplemented with 0.05% paraminobenzoic acid for emerging adults. On day 6–8 post-emergence, female mosquitoes were removed from emergence cages and transferred to 32 × 1.5L experimental cages, each containing 75–80 individuals. Each experimental cage contained females randomly chosen from each of the 3 emergence cages and mosquitoes were subsequently housed in a 19°C incubator (humidity 50 ± 5%). All mosquitoes were starved for 24 h before blood meals and given 30 min to feed. Of the 32 experimental cages, the mosquitoes in 12 were exposed to RD parasites, 12 to HD parasites and 8 to uninfected mice (control). Hydric stress and nutrient depletion have previously been found to be important in determining the virulence of malaria infection to mosquitoes (Ferguson and Read 2002a; Lambrechts et al. 2006; Aboagye-Antwi et al. 2010), so half of the experimental cages for each parasite line (*n* = 6 per condition) and control feeds (*n* = 4 per condition) were kept in ‘standard’ conditions with *ad libitum* access to 10% glucose and water solution and half were kept under ‘restricted’ conditions, where they only had access 50% of the time (following Ferguson and Read 2002a). To prevent mosquito mortality from drowning and to control access to water, mosquitoes were not provided with oviposition pools. In total, 32 experimental cages with 2187 mosquitoes contributed data to the analyses. This number of replicate cages for each combination of parasite line and environmental conditions is necessary because mosquitoes sharing a cage do not provide statistically independent data points.

### Generation of different parasite densities

Our experiment required that the RD and HD lines generated variation in the intensity of infections in mosquitoes and we validated this by measuring the densities of ookinetes and oocysts. Ookinete density inside blood meals is difficult to assess accurately. Therefore, we measured ookinete densities in *in vitro* cultures that mimic vector conditions (the standard way to measure parasite fertilization rates and ookinete production (Janse et al. 1985; Reece et al. 2008; Ramiro et al. 2011)). Cultures were set up from mice infected with either the RD (5 replicate infections) or HD lines (using the HD clone, PbMC1521 cl1 in 6 replicate infections; following the methods in Pollitt et al. 2010). To assess oocyst density, mosquitoes were infected as described above and 10 mosquitoes were randomly selected and removed from each cage on day 14 post-infection. Mosquitoes were anaesthetized with chloroform, dissected to extract midguts, and the infection status (positive or negative) and the number of oocysts per midgut were recorded. Equivalent numbers of mosquitoes from control cages were removed and discarded.

### Vector mortality and parasite proliferation

Testing the impact of parasite density on transmission potential requires quantifying the prevalence of sporozoite positive mosquitoes, the density of sporozoites in salivary glands and the survival of mosquitoes. On day 21 post-infection, we randomly selected 10 mosquitoes per cage to determine infection prevalence and the density of sporozoites in the salivary glands. Sporozoite presence and density was quantified by homogenizing salivary glands in 20 μL PBS before counting sporozoites on a haemocytomer. The prevalence of sporozoites was calculated as the proportion of mosquitoes dissected from each cage with detectable sporozoites. Parasite proliferation was calculated as the number of salivary gland sporozoites per infected mosquito. Equivalent numbers of mosquitoes from control cages were removed and discarded. To estimate vector mortality rates, cages were checked every second day, throughout sporogony (days 0–21) and then for a further 30 days. All dead mosquitoes were counted and removed. The mosquitoes sampled for counts of oocysts or sporozoites and the few remaining alive on day 50 (< 6 per cage) were treated as censored data points in the mortality analysis. One cage (RD line under restricted conditions) was removed from the survival analysis due to missing individuals.

### Statistical analyses

All analyses were performed using R version 2.12.1 (http://www.R-project.org). Infection prevalence at both oocyst and sporozoite stages and gametocyte sex ratios were analysed using generalized linear models with binomial error structures. Gametocyte density of the two lines was compared with a general linear model. Survival analysis was performed using Cox proportional hazard mixed effect models with experimental cage fitted as a random effect to overcome pseudoreplication problems of sampling multiple mosquitoes from each cage (Terry Therneau (2009) coxme: Mixed Effects Cox Models. R package version 2.0.). The differences in ookinete, oocyst and sporozoite (log transformed to meet normality assumptions) densities between the two lines were compared using linear mixed effect models with mouse (ookinete data) or cage (for oocyst and sporozoite data) fitted as random effects. To examine the effect of oocyst density on mosquito survival and parasite proliferation, we compared the mean oocyst densities per cage with the proportion of mosquitoes infected with sporozoites and the proportion of mosquitoes surviving to day 21 post-infection using generalized linear models with binomial error structures. In addition, we analysed the relationship between mean oocyst and mean sporozoite density per cage with a general linear model on log transformed data. With the exception of the survival analysis and the binomial generalized linear models, where the model output is reported, we followed model simplification by sequentially dropping the least significant term and comparing the change in deviance with and without the term to chi-square distributions until the minimal adequate model was reached. Degrees of freedom correspond to the difference in the number of terms in the model.

## Results

### Generation of different parasite densities

**Figure 1:**
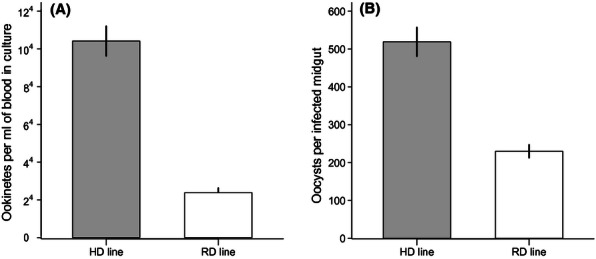
Generation of different infection densities. Mean ± SE Density of HD and RD lines for (A) ookinete (based on between 5 and 6 replicate infections and between 20 and 24 cultures per line) and (B) oocyst (based on 12 cages per line with 10 mosquitoes per cage) stage parasites.

The HD line generated more than four times the mean number of ookinetes than the RD line (ookinetes per mL of infected blood: RD = 23 800 (± 2466 SE); HD = 104 167 (± 7877 SE); *χ*^2^_1_ = 16.045, *P* < 0.0001; Fig. 1A). This difference is partly explained by slightly higher gametocyte densities in HD than RD infections in mice used for setting up cultures (mean number of gametocytes per mL of blood: RD = 1.10 × 10^10^ (± 8.57 × 10^8^ SE); HD = 1.86 × 10^10^ (± 1.14 × 10^9^ SE); *χ*^2^_1_ = 5.81, *P* = 0.016) and an almost two-fold greater fertilization rate in the HD line (ookinete density/female gametocyte density: RD = 3.5 × 10^−3^ (± 4.5 × 10^−4^ SE); HD = 6.6 x 10^-3^(± 4.4 × 10^−4^ SE); *χ*^2^_1_ = 6.436, *P* = 0.0112).

The proportion of mosquitoes infected with oocysts was high (>81% (± 4 SE)) and was not significantly affected by either the conditions the mosquitoes experienced (standard vs restricted; *χ*^2^_1_ = 0.074, *P* = 0.39), or the parasite line (*χ*^2^_1_ = 1.54, *P* = 0.22). Oocyst density was also unaffected by the conditions mosquitoes were kept under (*χ*^2^_1_ = 0.0002, *P* = 0.99), but infection with the HD line resulted in more than twice as many oocysts per infected mosquito (RD = 230 (± 17 SE); HD = 519 (± 38 SE); *χ*^2^_1_ = 27.19, *P* < 0.0001; Fig. 1B). This difference is not explained by variation across the lines in either the sex ratio or density of gametocytes in the infections mosquitoes fed on (proportion male *χ*^2^_1_ = 0.158, *P* = 0.69; gametocytes per mL of blood *χ*^2^_1_ = 0.499, *P* = 0.50).

### Parasite proliferation

**Figure 2:**
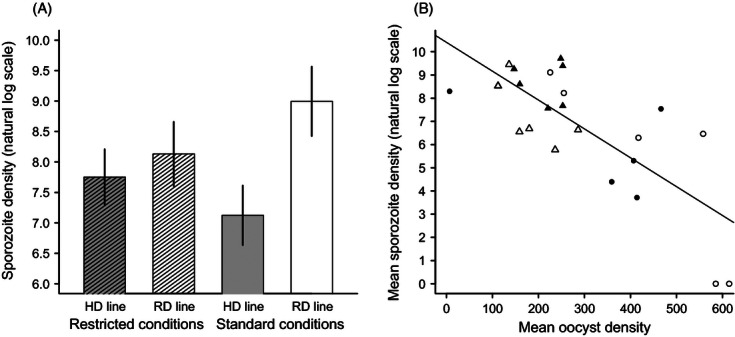
Parasite proliferation reduced at high densities. Log sporozoite density per mosquito at day 21 shown as: (A) mean ± SE per density treatment and condition (based on 6 replicate cages with 10 mosquitoes dissected per cage), or (B) in relation to mean oocyst density for per cage. Each point represents one cage of mosquitoes with 10 individuals dissected per cage at each stage for regular density line (triangles) or the high density line (circles) under either standard conditions (solid symbols) or restricted conditions (open symbols). The line shows the predicted relationship from the minimal model.

Having confirmed that the HD line resulted in significantly higher numbers of ookinetes and oocysts than the RD line, we then examined parasite proliferation, measured by the density of sporozoites successfully reaching the salivary glands. As parasite line had a borderline significant effect on the density of sporozoites in salivary glands (Table 1), we examined line effects in each of the two conditions separately. One cage (HD standard conditions) was excluded from the proliferation analysis because only one of the ten mosquitoes dissected at day 14 was infected, and only with a single oocyst, suggesting that the infection failed to transmit from this host. In standard conditions, the HD line produced significantly fewer sporozoites per mosquito than the RD line but under restricted conditions, there was no significant effect of parasite line, and sporozoite densities were intermediate between the RD and HD infections in standard conditions (Table 1, Fig. 2A). As these results suggested that, under standard conditions, there is a negative effect of high oocyst numbers on parasite proliferation, we examined this in more detail by comparing the mean densities of oocysts and sporozoites for each cage. Across all treatment groups, mean sporozoite density was significantly and negatively associated with mean oocyst density, but there were no additional effects of line or condition (Table 1, Fig. 2B). The prevalence of mosquitoes infected with salivary gland sporozoites at day 21 was significantly lower for mosquitoes fed on the HD line than the RD line (Table 1). This relationship was driven by a strong negative correlation between the mean oocyst density per cage and the proportion of mosquitoes infected with sporozoites (Table 1). When mean oocyst density was included in the model there was no additional effect of line or condition (standard vs restricted; Table 1).

**Table 1:** Density effects on parasite proliferation

Line and condition effect on presence and density of salivary gland sporozoites (day 21)				
		Mean (± SE)		
Sporozoites per mosquito (log)	All data		Condition *χ*^2^_1_ = 0.076, *P* = 0.78	
			**Parasite line** ***χ***^**2**^_**1**_ **= 5.29,** ***P*** **= 0.021**	
			Line × condition *χ*^2^_1_ = 0.090, *P* = 0.76	
	Restricted conditions			
	RD	8.13 (± 0.53)	Parasite line *χ*^2^_1_ = 2.60, *P* = 0.12	
	HD	7.75 (± 0.46)		
	Standard conditions			
	RD	9.00 (± 0.57)	**Parasite line** ***χ***^**2**^_**1**_ **= 4.83,** ***P*** **= 0.028**	
	HD	7.12 (± 0.49)		
Prevalence (proportion of Mosquitoes infected with sporozoites)				
	RD	46% (± 4.6)	**Parasite line Z = 2.27,** ***P*** **= 0.022**	
	HD	30% (± 4.2)	Condition Z = 0.30, *P* = 0.76	
Relationship between mean oocyst density and the mean sporozoite density and prevalence of sporozoites per cage				

		Intercept	Slope
Mean sporozoite density (log)	Parasite line *χ*^2^_1_ = 0.99, *P* = 0.33	**10.41**	**−0.124**
	Condition *χ*^2^_1_ = 0.18, *P* = 0.67		
	**Mean oocyst density** ***χ***^**2**^_**1**_ = **26.18,** ***P*** **< 0.0005**		
Sporozoite prevalence	Parasite line Z = 0.30, *P* = 0.77	**0.48**	**−0.003**
	Condition Z = 0.03, *P* = 0.98		
	**Mean oocyst density Z = 2.66,** ***P*** **< 0.001**			

Statistically significant results highlighted in bold.

### Vector survival

#### Survival during sporogony

**Figure 3:**
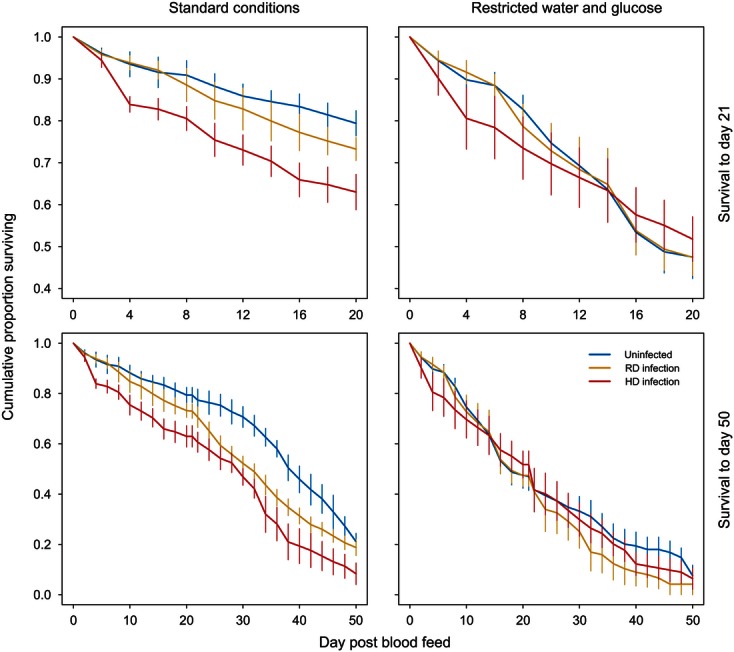
The effect of parasite line and environmental conditions on vector survival. Cumulative proportion of mosquitoes surviving after receiving a control uninfected blood meal (blue), infection with the regular density line (RD; yellow) or the high density line (HD; red) under standard or restricted glucose and water conditions. Top panels show survival up to day 21 (when the sporozoites reach the salivary glands) and bottom panels show the full 50 days over which mortality was recorded. Each point represents the mean survival for between 4 and 6 cages and the error bars show the standard error of the mean.

Under standard conditions, mosquitoes infected with the HD line were significantly less likely to survive to day 21 than both uninfected and RD infected mosquitoes, which did not differ significantly in survival (Table 2, Fig. 3). Keeping mosquitoes under restricted conditions significantly reduced survival during sporogony (Coxme: dead = 878, total = 2187, Z = 4.91, *P* < 0.00001; Fig. 3) similarly across control, RD and HD infected mosquitoes. In addition, across both conditions there was a significant negative correlation between the mean oocyst density per cage and the proportion of mosquitoes surviving to day 21 (Table 2).

**Table 2:** The effect of parasite line (High density (HD) and regular density (RD)) and mean oocyst density on mosquito lifespan

		*n* dead, *n* total	Coxme analysis
Standard conditions			
Survival to day 21	**HD** vs **control**	**218, 710**	**Z = 3.26,** ***P*** **= 0.001**
	**HD** vs **RD**	**276, 841**	**Z = 2.08,** ***P*** **= 0.037**
	RD vs control	176, 701	Z = 1.46, ***P*** = 0.15
Survival to day 50	**HD** vs **control**	**450, 710**	**Z = 4.09,** ***P*** **< 0.0001**
	HD vs RD	539, 841	Z = 1.77, ***P*** = 0.077
	**RD** vs **control**	**437, 701**	**Z = 2.53,** ***P*** **= 0.011**
Restricted conditions			
Survival to day 21	HD vs control	359, 710	Z = 0.05, ***P*** = 0.96
	HD vs RD	395, 776	Z = 0.17, ***P*** = 0.86
	RD vs control	332, 636	Z = 0.11, ***P*** = 0.91
Survival to day 50	HD vs control	498, 710	Z = 0.48, ***P*** = 0.63
	HD vs RD	539, 776	Z = 0.41, ***P*** = 0.68
	RD vs control	439, 636	Z = 0.67, ***P*** = 0.5
Relationship between mean oocyst density per cage and proportion of mosquitoes alive on day 21			

			Effect size
Proportion of mosquitoes alive on day 21	Parasite line Z = 0.77, df = 1, ***P*** > 0.5		
	**Mean oocyst density Z = 2.31, df = 1,** ***P*** **= 0.021**		**−0.0008**
	**Condition Z = 7.53, df = 1,** ***P*** **< 0.0001**		**−0.85**

Significant effects are highlighted in bold.

#### Longer term survival

Most studies examining the effect of malaria infection on mosquito survival only follow the vectors to the point where transmission can begin (i.e., when sporozoites reach the salivary glands). However, as sporozoites remain in the salivary glands (Shute 1945; Dawes et al. 2009b) the longer a mosquito survives, the more infectious bites it can make (termed the longevity factor or expected infective life (Garret-Jones 1964)). Therefore, we continued to measure vector mortality until day 50 (when less than 2% of all infected and uninfected mosquitoes remained alive). Survival (from day 0 to 50) under standard conditions was significantly lower for infected compared with control mosquitoes. In addition, there was a borderline non-significant trend where survival of mosquitoes infected with the HD line was lower than for the RD line (Table 2; Fig. 3). As with survival during sporogony, keeping mosquitoes under restricted conditions significantly reduced longer term survival (Coxme: dead = 1451, total = 2187, Z = 5.70, *P* < 0.00001; Fig. 3) similarly across control, RD and HD infected mosquitoes (Table 2).

### Lifetime contribution of mosquitoes to transmission

Our data demonstrate that parasites in high-density infections suffer from crowding in terms of reduced parasite proliferation and increased vector mortality. To estimate the cumulative impact of these different density-dependent processes on transmission potential, we extended the mathematical model developed in Churcher et al. (2010; see Data S1 for model details) and parameterized it using data from our experiment. Here, ‘relative transmission’ is defined as the total number of sporozoites available to infect a vertebrate host during the lifetime of the vector. It is calculated by multiplying the number of infectious bites made by a mosquito that becomes infected during its first blood meal by the number of salivary gland sporozoites it harbours (Fig. S1). This method provides a relative estimate of overall transmission that takes into account both the presence and density of sporozoites within the salivary glands (however, see Churcher et al. 2010 for discussion of the frailties of this metric).

**Figure 4:**
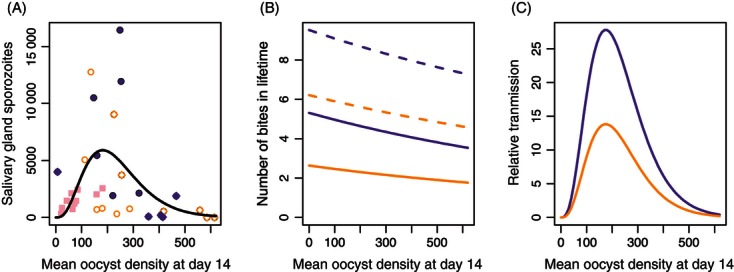
Using a mathematical model to quantify the cumulative impact of density-dependent parasite development (A) and vector mortality (B) on overall transmission (C). (A) The fitted relationship between oocyst density and the number of sporozoites in the salivary glands (solid black line). Open orange circles are cages under restricted conditions, purple filled circles are cages under standard conditions and pink squares denote data from (Sinden et al. 2007). (B) The change in the number of bites (dashed line) and infectious bites (solid line) during the lifetime of the mosquito. (C) The overall impact on transmission as defined as the number of parasites available to establish a new infection weighted by the number of hosts bitten. Colours in (B) and (C) denote standard (purple) and restricted (orange) mosquito conditions.

Previous experiments using the same parasite-vector combination – but at a lower infection intensities – had suggested that the relationship between the mean number of oocysts and the mean number of salivary gland sporozoites is best described by a hyperbolic function (Sinden et al. 2007). Our analysis revealed that the number of salivary gland sporozoites produced per oocyst decreases at high oocyst densities. Therefore, to estimate productivity across a wider range of oocyst densities, we combined data from Sinden et al. (2007) with the data generated in our study from both RD and HD infections. We used data from Sinden et al. (2007) because density counts are available for individual mosquitoes. Because environmental conditions did not significantly influence oocyst density, we fitted a single curve to all data using methods described in Sinden et al. (2007), but using a gamma function to describe the relationship between salivary gland sporozoite density and oocyst load to capture its humped (as opposed to saturating) shape (Fig. 4A). The total (net) number of sporozoites produced per mosquito, peaks in the middle of the the range of oocyst densities that were tested. The model fitting process is described in the supporting information. We quantified the change in vector survival resulting from variation in parasite density using methods and functional forms described in Dawes et al. (2009a). Our data show that vector survival is strongly influenced by environmental conditions (Fig. 3), so we fitted different curves for restricted and standard conditions (Fig. 4B). Finally, by multiplying the relationships for parasite productivity (Fig. 4A) and the potential number of infectious bites (Fig. 4B) we calculated a relative transmission index, which describes the number of parasites available to establish a new infection weighted by the potential number of hosts bitten. Supporting previous work, our model predicts that at low infection intensities, an increase in parasite density results in higher potential transmission for mosquitoes in both standard and restricted conditions. However, our model also predicts that as infection intensity increases beyond an optimal oocyst burden (around 200 oocysts per mosquito in our system), negative density dependence reduces overall transmission potential (Fig. 4C).

## Discussion

Through a combination of experimental data and mathematical modelling, we show that density-dependent processes can significantly influence parasite transmission potential. Our data reveal that the costs of crowding manifest in two ways. First, high-density infections reduce the proliferation of malaria parasites inside the vector (fewer sporozoites reach the salivary glands leading to both a lower prevalence of sporozoite infected mosquitoes and lower sporozoite densities in those infected). Second, in controlled laboratory conditions, high-density infections result in fewer vectors surviving until parasites have completed development (sporogony). The second effect also impacts upon vector fitness because high-density infections are more likely to kill mosquitoes earlier in their life. Furthermore, the consequences of high-density infections for vectors and parasites depend on environmental conditions (sugar limitation and hydric stress). Previous studies have suggested that the relationship between the densities of oocysts on the midgut and salivary gland sporozoites, in individual mosquitoes, saturates (Sinden et al. 2007; Churcher et al. 2010). However, by extending the range of oocyst densities examined, we show that the relationship is humped, with a decrease in sporozoite production at high oocyst densities. Data on the relationships between the number of sporozoites in the salivary glands, the number injected per bite and the probability of establishing an infection in the vertebrate host are scarce but suggest positive trends between these factors (Kebaier et al. 2009 but see Beier 1993 for a discussion of complications). Therefore, our epidemiological model predicts that the maximum potential for transmission is reached at an intermediate value of the parasite densities examined. Specifically, our model predicts that transmission from mosquitoes in *ad libitum* conditions is more than double that from mosquitoes experiencing sugar restriction and hydric stress, but the optimal oocyst density is equivalent (∼200 for our system) in both environmental conditions. In the extreme, our experimental data show that doubling the number of oocysts from the mean of 250 resulted in 85% fewer salivary gland sporozoites under standard environmental conditions.

A number of non-mutually exclusive factors could drive density dependence in parasite proliferation within the vector. Resources that are needed for parasite development, such as nutrients and space on the midgut, could limit the number of sporozoites produced per oocyst in a density-dependent manner. In addition, mosquitoes could mount stronger immune defences in response to high-density infections (Mendes et al. 2011). This includes melanization of oocysts or of sporozoites as they travel to the salivary glands (Cirimotich et al. 2010). Although it is not possible to determine the relative contributions of resource- and immune-mediated factors in our data, resource limitation in the production of sporozoites could explain why our regular density line did not produce significantly more sporozoites under restricted conditions. The interactions between parasite density, immunity and resource availability should become testable thanks to the growing tool set for quantifying and manipulating insect immune responses (Tripet et al. 2008; Cirimotich et al. 2010). Density dependence in parasite proliferation also has implications for the evolution of virulence (when virulence results from high a replication rate) because the death of the host/vector is not the only constraint on virulence.

Our survival data suggest that, in this laboratory system, mosquito mortality increases when mosquitoes are infected with high parasite densities at the oocyst stage (HD line). Although some studies have suggested that mosquito survival is reduced by infection with malaria parasites (see Ferguson and Read 2002b; Dawes et al. 2009a; Aboagye-Antwi et al. 2010), others have not detected an effect on mosquito lifespan (e.g. Robert et al. 1990; Gamage-Mendis et al. 1993). These contradictory data could be explained if the cost of infection is only detectable under certain conditions (Tripet et al. 2008; Vézilier et al. 2012). Furthermore, the costs of malaria infection to mosquitoes may be further complicated by the recent discovery that ookinetes can undergo apoptosis in the blood meal (a form of programmed cell death (Al-Olayan et al. 2002; Arambage et al. 2009; Pollitt et al. 2010)) instead of traversing the midgut wall to form oocysts. Apoptosis has been proposed as a parasite strategy to regulate parasite density in the vector when co-infecting parasites are closely related (Al-Olayan et al. 2002), but its role in infections is poorly understood (Pollitt et al. 2010; Reece et al. 2011). Under both environmental conditions, mosquitoes infected with the HD line experienced high mortality in the first 4 days post-infection. This is consistent with previous studies showing that epithelium cells are killed when ookinetes traverse the midgut, and although the epithelium repairs and seals (Han and Barillas-Mury 2002), high densities of ookinetes may lead to permanent damage or increase the risk of secondary bacterial infections (Dimopoulos et al. 2002; Rodrigues et al. 2010). Furthermore, immune responses stimulated by high-density infections could cost resources and/or result in immunopathology (Sadd and Siva-Jothy 2006). In addition, mosquitoes are likely to suffer an energy cost of high-density infections, which is most likely to be paid whilst parasites are rapidly replicating inside oocysts (Carwardine and Hurd 1997). More broadly, our data show that infected mosquitoes may experience increased mortality when compared with uninfected controls. This supports the hypothesis that malaria can be harmful for mosquitoes (Ferguson and Read 2002b) but also shows that detecting this effect will depend on environmental conditions.

Reducing vector lifespan can have considerable effects on transmission because the development time required for malaria parasites to colonize the salivary glands (∼3 weeks for *P. berghei*; Sinden et al. 2007) is long compared to the average life expectancy of mosquitoes in the wild, which is estimated as low as 1–2 weeks for adult females of the *Anopheles* complex (Gwadz and Collins 1996). In addition to reducing parasite fitness, high parasite densities are also likely to reduce mosquito reproductive output because their mortality was greatest in the first few days after blood feeding. Our model also predicts the impact of density-dependent parasite-vector interactions is shaped by environmental conditions. Under natural settings, mosquitoes are likely to experience varying levels of hydric and nutrient stress depending on habitat and season (Tripet et al. 2008). Restricting sugar and water reduced vector survival during sporogony, and whilst the effects of parasite density on sporozoite production and vector survival are not as strong under restricted conditions, the patterns are qualitatively similar to *ad libitum* conditions. The impact of vector environment on parasite density-dependent relationships may be complex because nutrient limitation may reduce the resources available for parasite development as well as for vectors to mount an immune response (Schmid-Hempel 2005; Tripet et al. 2008). Furthermore, the combined effect of nutrient and hydric stress on mosquito mortality may alone exceed the costs of infection. Our results demonstrate the importance of environmental variation in determining parasite dynamics and interactions with the vector, and the next challenge will be incorporating the effects of other important environmental factors (e.g. temperature (Lambrechts et al. 2011; Paaijmans et al. 2012)) into transmission patterns.

Model systems provide a powerful tool to study parasite dynamics, but it should be noted that the oocyst densities examined here are substantially higher than those generally reported for wild caught mosquitoes infected with human malaria parasites (e.g. Vaughan et al. 1992 but see White et al. 2011). Also, the interactions between rodent malaria parasites and *Anopheles stephensi* may differ from parasite-vector interactions in natural infections with human malaria parasites (Aguiler et al. 2005; Cohuet et al. 2006; Tripet et al. 2008). However, if the shape of the relationship observed here reflects natural infections, our results would have important implications for the development of transmission-blocking interventions (TBIs). Potential targets for TBIs are generally evaluated in terms of reductions in oocyst number rather than the prevalence of infected mosquitoes (e.g. Miura et al. 2007; Chowdhury et al. 2009; see Churcher et al. 2012 for discussion). However, an intervention that reduces oocyst density may mitigate the cost of crowding enhancing both vector survival and sporozoite production. There is a clear need to quantify density-dependent relationships for the range of densities observed in natural infections because models predict that even slight variations in vector survival and sporozoite production have significant effects on parasite transmission (Macdonald 1957; Koella 1999).

Furthermore, understanding density dependent relationships in malaria parasites will be more complicated if ookinetes undergo apoptosis to regulate the number of oocysts (Al-Olayan et al. 2002; Pollitt et al. 2010; Reece et al. 2011) because the relatedness between co-infecting parasites will also need to be considered (West et al. 2006). The occurrence of, and putative altruistic explanation for, apoptosis in protozoan parasites is controversial, but for apoptosis to be an adaptive parasite strategy, a key requirement is that the surviving parasites benefit from density regulation. By revealing that parasites incur fitness costs in high-density infections, our data support the hypothesis that natural selection favours parasite genotypes that actively regulate their density to avoid these costs. Data from previous studies suggest that parasites invest in sexual stages and adjust their sex ratios in a density-dependent manner (Reece et al. 2008; Pollitt et al. 2011), but whether parasites can detect density and adjust their development accordingly in the vector remains unknown.

In conclusion, we demonstrate that rodent malaria parasites in high-density infections experience crowding, which reduces parasite productivity and increases the risk of vector mortality before parasites complete development and have the potential for onwards transmission. Our model suggests that interactions between density-dependent processes and environmental conditions can have considerable effects on the transmission of vector-borne diseases. Understanding how these processes influence transmission potential in natural settings and parasite-vector combinations is crucial for the success of control strategies.

## Data archiving statement

Data for this study are available at Dryad**:** doi:10.5061/dryad.44jf2.

## Supporting Information

**Data S1.** Description of model structure for the lifetime contribution of mosquitoes to transmission.
