# Supplementary material for: Costs of crowding for the transmission of malaria parasites
Source: Evol Appl. 2013 Feb 11;6(4):617–29. doi: 10.1111/eva.12048 (PMC3684743; doi:10.1111/eva.12048)
Supplement: Supplementary file 1 [file eva0006-0617-SD1.docx]

**S1: Description of model structure for the lifetime contribution of mosquitoes to transmission**

**Figure s1:** Simplified model structure

The model tracks the number of *Plasmodium* parasites through their progression from oocysts at day 14 to sporozoites at day 21 as described by Churcher *et al.* (2010). The individual-based model tracks the number of parasites in each mosquito as to overcome problems associated with mean-based models that typically underestimate the cumulative impact of multiple density-dependent processes. The number of salivary gland sporozoites that develop in mosquito $i$, denoted $y_{i}$, is randomly selected from a negative binomial distribution using the following equation,

$y_{i}\sim\text{NegBin}\left[f\left(x_{i}\right),k_{i}\left(y_{i}\right)\right]$ [1]

$k_{i}\left(y_{i}\right)=\frac{\kappa_{1}y_{i}}{1+\kappa_{2}y_{i}}$ [2]

where $x_{i}$ mean number of oocysts per mosquito at day 14, $k_{i}\left(y_{i}\right)$ is the overdispersion parameter of the negative binomial distribution of parasite life-stages among mosquitoes, which itself depends on sporozoite density as described in (Sinden *et al.* 2007). The overdispersion parameter is an inverse function of the degree of parasite aggregation such that very small values correspond to a highly overdispersed distribution and values greater than 5 tend to a random, Poisson distribution. The function $f\left(x_{i}\right)$ determines the relationship between the mean number of oocysts at day 14 and the mean number of salivary gland sporozoites at day 21. A variety of functions were tested for this relationship (outlined in Sinden *et al.* 2007) and the most parsimonious was the gamma function which is described by the following equation,

$f_{i}\left(x_{i}\right)=\frac{1}{\alpha}\beta^{\gamma}x_{i}^{\left(\gamma-1\right)}\exp\left(-\frac{x}{\alpha}\right)$. [3]

Parameters $\alpha$, $\beta$ and $\gamma$ were estimated by fitting equation [3] to data presented in Figure 4a using maximum likelihood. The transition from oocyst to sporozoites cannot be measured directly for individuals because mosquitoes are fatally damaged during the oocyst counting procedure, therefore cage means are taken as units of replication. The function gives a gamma shaped curve which captures a process by which increasing parasite numbers initially facilitates but later restricts population size. A full list of parameter definitions and values (and their 95% confidence intervals) are given in Table S1.

**Effect of oocyst density on vector mortality.** The survival function of a mosquito at time $t$ after the cessation of first engorgement is described (from Dawes *et al.* (2009)) by,

$\lambda_{i}\left(t,x_{i}\right)=\exp\left\{-\left[\left(\nu_{0}+\nu_{1}x_{i}\right)\frac{t^{3}}{3}-\left(\delta_{0}+\delta_{1} x_{i}\right)\frac{t^{2}}{2}+\left(\theta_{0}+\theta_{1} x_{i}\right)\right]\right\}$ [4]

where $x_{i}$ is oocyst density and the expression within the square brackets is the parabolic hazard that best fitted the instantaneous mosquito mortality rate data as a function of time post-feeding and parasite density in Dawes *et al.* (2009). Parameter values were estimated by re-fitting the hazard contained in equation [4] to our original mortality data. Integrating $\lambda_{i}\left(t,x_{i}\right)$ with respect to time gives the life-expectancy of mosquito $i$at cessation of engorgement, $\Lambda_{i}\left(x_{i}\right)$.

For simplicity we assume that all sporozoites are within the salivary glands $n$ days after feeding. If mosquitoes bite once every $1/a$ days then the number of potentially infectious bites made in the lifetime of mosquito $i$,$\Phi_{i}\left(x_{i}\right)$, is given by,

$\Phi_{i}\left(x_{i}\right)=\left\{\begin{matrix} 0 \Lambda_{i}\left(x_{i}\right)<n \\ \left[\Lambda_{i}\left(x_{i}\right)-n\right]a \Lambda_{i}\left(x_{i}\right)\geq n \end{matrix}\right.$. [5]

**Relative transmission**

The mean number of salivary gland sporozoites available to be injected over the lifetime of a mosquito infected during its first bloodmeal, $T$, is

$T=\sum_{i=1}^{r} \Phi_{i}\left(x_{i}\right)y_{i}/r$ [6]

where $r$is the number of mosquitoes in the population (which is given a value of 100,000 to reduce the impact of stochastic fluctuations). This ‘relative transmission’ index assumes that number of sporozoites injected by a mosquito during blood feeding is relatively small in comparison with the number in the salivary glands (and therefore $y_{i}$remains constant over successive bites). For a full description of the metric see Churcher *et al.* (2010). **Table S1. Parameter values and definitions.** Values are taken from the data collected in the experiments presented in this paper and Churcher *et al. (*2010) unless otherwise stated.

| Symbol | Description | Equation and values |
| --- | --- | --- |
| $x_{i}$ | number of oocysts on day 14 in mosquito $i$ | - |
| $y_{i}$ | number of salivary gland sporozoites on day 21in mosquito $i$ | equation [1] |
| $f\left(x_{i}\right)$ | function describing the number of salivary gland sporozoites developing within mosquito $i$ from oocysts | equation [3] $\alpha=55.3$ $\beta=4.80\times10^{9}$ $\gamma=4.28$ |
| $k_{i}\left(x_{i}\right)$ | distribution of oocysts within the mosquito population | equation [2] $\kappa_{1}=62.2$ $\kappa_{2}= 0.018$ |
| $\lambda_{i}\left(t,x_{i}\right)$ | survivorship function of mosquito $i$ at time $t$. | equation [4]  $\nu_{0}=-0.966\times10^{-5}$;$\nu_{1}=2.99\times10^{-7}$ $\delta_{0}=5.11\times10^{-3}$;$\delta_{1}=-1.19\times10^{-5}$ $\theta_{0}=3.56\times10^{-3}$;$\theta_{1}=1.06\times10^{-4}$ |
| $\Lambda_{i}\left(x_{i}\right)$ | life expectancy of mosquito $i$ after the cessation of engorgement | $\Lambda_{i}\left(x_{i}\right)=\int_{t=0}^{\infty} \lambda_{i}\left(t,x_{i}\right)dt$ |
| $a$ | biting rate per mosquito on humans | 0.3 day^–1*^ |
| $n$ | mean duration of the extrinsic incubation period within mosquito | 16 days^†^ |
| $r$ | number of mosquitoes in the population | 100,000 |
| $\Phi_{i}$ | number of potentially infectious bites made by mosquito $i$ during its lifetime | equation [5] |
| $T$ | mean number of infectious bites made by the mosquito population over the lifetime of a mosquito infected during its first bloodmeal | equation [6] |

^*^ from Smith & McKenzie 2004

^†^ unpublished results for the mean latent period of the parasite-vector combination at 19^o^C (the temperature our own studies were conducted at)

**References**

Churcher, T.S., E.J. Dawes, R.E. Sinden, G. Christophides, J. Koella, and M.G. Basáñez, M.G. 2010. Population biology of malaria within the mosquito: density-dependent processes and potential implications for transmission-blocking interventions. Malaria Journal 9:311.

Dawes, E.J., T.S. Churcher, S. Zhuang, R.E. Sinden, and M.G. Basáñez. 2009. *Anopheles* mortality is both age- and *Plasmodium*-density dependent: implications for malaria transmission. Malaria Journal 8:228.

Sinden, R.E., E.J. Dawes, Y. Alavi, J. Waldock, O. Finney, J. Mendoza, G.A. Butcher, L. Andrews, A.V. Hill, S.C. Gilbert, and M.G. Basáñez. 2007. Progression of *Plasmodium berghei* through *Anopheles stephensi* is density-dependent. PLoS Pathogens 3:e195.

Smith, D.L., and F.E. McKenzie. 2004. Statics and dynamics of malaria infection in Anopheles mosquitoes. Malaria Journal 3:13
